# Supplementary material for: Therapeutic Potential of Bee and Wasp Venom in Anti-Arthritic Treatment: A Review
Source: Toxins (Basel). 2024 Oct 22;16(11):452. doi: 10.3390/toxins16110452 (PMC11598298; doi:10.3390/toxins16110452)
Supplement: Supplementary file 1 [file toxins-16-00452-s001.zip › toxins-3202952-supplementary.pdf]

Therapeutic Potential of Bee and Wasp Venom in Anti-Arthritic Treatment: A Review

Table S1. The Summary Table of Bee (*Apis*) Venom Protein and Peptide Constituents.

	Entry	Protein names	Length	Pharmacological effect	Organism
1	P01500	Apamin (APM) (Apamine)	46	Toxin with unique selectivity to KCa2 channels; Potently blocks human, rat and mouse KCa2.2/KCNN2/SK2 channels; In vivo, intracerebroventricular injection into rats of a dose of 1 ng results in neurodegeneration specifically in the Purkinje cells of the cerebellum, and induces seizures characterized by hypersensitivity to noise, loss of postural control, paroxystic jerking, and alternating periods of great agitation with tonic-clonic convulsions and periods of total prostration; When administered at high doses, exerts anti-inflammatory, anti-oxidative, anti-fibrotic and anti-apoptotic properties in several models of inflammatory disease, including gouty arthritis, atherosclerosis, atopic dermatitis and acute kidney injury; Down-regulates pro-inflammatory signaling pathways, such as the NF-κB and STAT3 pathways, probably by blocking SK channels such as KCa2.2/KCNN2/SK2 and/or KCa2.3/KCNN3/SK3 which are thought to be involved in promoting some inflammatory responses; or example in mouse and rat microglia cells, inhibits LPS-activated KCa2.2/KCNN2/SK2 channels and TLR4 expression leading to the down-regulation of the NF-kappaB, STAT, and MAPK/ERK signaling pathways and, as a	*Apis mellifera* (Honeybee)

				consequence, decreases secretion of pro-inflammatory cytokines.	
2	P83563	Allergen Api m 6.03 / Api m 6.04 (allergen Api m 6) [Cleaved into: Allergen Api m 6.01 / Api m 6.02]	92		*Apis mellifera* (Honeybee)
3	A0A8U0WQG8	Apyrase (EC 3.6.1.5)	567		*Apis mellifera* (Honeybee)
4	P0DQX7	Antimicrobial peptide Xac-3 (Xac3)	19	Antimicrobial and mast cell degranulating peptide which probably acts by forming pores in membranes (By similarity).<br>Active against both Gram-negative and Gram-positive bacterial strains as well as against yeasts (By similarity).<br>Has little hemolytic activity (By similarity).<br>In the context of inflammation and cancer tests, is weakly cytotoxic to normal cells, induces calcium signaling but does not impact cAMP production.<br>In addition, prevents LPS-induced nitric oxid (NO) synthesis but does not affect the IP3 signaling and pro-inflammatory activation of endothelial cells.<br>Does not show significant antiproliferative activity on the breast cancer cell line MDA-MB-231.	*Xylocopa violacea* (Violet carpenter bee) *(Apis violacea)*
5	P0DQX8	Antimicrobial peptide Xac-4 (Xac4)	19	Antimicrobial and mast cell degranulating peptide which probably acts by forming pores in membranes (By similarity).<br>Active against both Gram-negative and Gram-positive bacterial strains as well as against yeasts (By similarity).<br>Has little hemolytic activity (By similarity).<br>In the context of inflammation and cancer tests, is weakly cytotoxic to normal cells, induces calcium signaling but does not impact cAMP production.	*Xylocopa violacea* (Violet carpenter bee) *(Apis violacea)*

				In addition, prevents LPS-induced nitric oxid (NO) synthesis but does not affect the IP3 signaling and pro-inflammatory activation of endothelial cells .<br>Also shows significant antiproliferative activity on the breast cancer cell line MDA-MB-231.	
6	Q86QT2	Apamin	46	Neurotoxin that blocks voltage-independent calcium-activated potassium channels (KCNN1=SK1, KCNN2=SK2, KCNN3=SK3).	*Apis cerana cerana* (Oriental honeybee)
7	A0A0I9RJ80	Antigen 5-like protein	202		*Apis mellifera* (Honeybee)
8	M1FPC1	Antigen 5-like protein	202		*Apis mellifera carnica* (Carniolan honeybee)
9	Q336K2	Acid phosphatase	156		*Apis mellifera* (Honeybee)
10	P07493	Bombolitin-2 (Bombolitin II)	17	Mast cell degranulating peptide.<br>Its mast cell degranulation activity may be related to the activation of G-protein coupled receptors in mast cells as well as interaction with other proteins located in cell endosomal membranes in the mast cells (By similarity).	*Bombus pensylvanicus* (American bumblebee) *(Apis pensylvanica)*
11	P07494	Bombolitin-3 (Bombolitin III)	17	Mast cell degranulating peptide.<br>Its mast cell degranulation activity may be related to the activation of G-protein coupled receptors in mast cells as well as interaction with other proteins located in cell endosomal membranes in the mast cells (By similarity).	*Bombus pensylvanicus* (American bumblebee) *(Apis pensylvanica)*
12	P07495	Bombolitin-4 (Bombolitin IV)	17	Mast cell degranulating peptide.<br>Its mast cell degranulation activity may be related to the activation of G-protein coupled receptors in mast cells as well as interaction with other proteins located in cell endosomal membranes in the mast cells (By similarity).	*Bombus pensylvanicus* (American bumblebee) *(Apis pensylvanica)*
13	P07496	Bombolitin-5 (Bombolitin V)	17	Mast cell degranulating peptide.<br>Its mast cell degranulation activity may be related to the activation of G-protein coupled receptors in mast cells as	*Bombus pensylvanicus* (American bumblebee) *(Apis pensylvanica)*

				well as interaction with other proteins located in cell endosomal membranes in the mast cells (By similarity).	
14	P0CD67	Bombolitin-6	19	Mast cell degranulating peptide. May also display antibacterial and antifungal activities (By similarity).<br>Its mast cell degranulation activity may be related to the activation of G-protein coupled receptors in mast cells as well as interaction with other proteins located in cell endosomal membranes in the mast cells (By similarity).	*Bombus lapidarius* (Red-tailed bumblebee) *(Apis lapidaria)*
15	P0CD68	Bombolitin-7	19	Mast cell degranulating peptide. May also display antibacterial and antifungal activities (By similarity).<br>Its mast cell degranulation activity may be related to the activation of G-protein coupled receptors in mast cells as well as interaction with other proteins located in cell endosomal membranes in the mast cells (By similarity).	*Bombus lapidarius* (Red-tailed bumblebee) *(Apis lapidaria)*
16	P0CD69	Bombolitin-8	19	Mast cell degranulating peptide. May also display antibacterial and antifungal activities (By similarity).<br>Its mast cell degranulation activity may be related to the activation of G-protein coupled receptors in mast cells as well as interaction with other proteins located in cell endosomal membranes in the mast cells (By similarity).	*Bombus lapidarius* (Red-tailed bumblebee) *(Apis lapidaria)*
17	P10521	Bombolitin-1 (Bombolitin I)	17	Mast cell degranulating peptide.<br>Its mast cell degranulation activity may be related to the activation of G-protein coupled receptors in mast cells as well as interaction with other proteins located in cell endosomal membranes in the mast cells (By similarity).	*Bombus pensylvanicus* (American bumblebee) *(Apis pensylvanica)*
18	A0A7M6W880	Bee-milk protein	423		*Apis mellifera* (Honeybee)
19	B3GM11	Bee-milk protein	415		*Apis mellifera* (Honeybee)
20	B3GM12	Bee-milk protein	422		*Apis mellifera* (Honeybee)
21	Q6TGR0	Bee-milk protein	416		*Apis mellifera* (Honeybee)

22	F1CGQ2	CLIP domain-containing serine protease (EC 3.4.21.-)	358		*Bombus terrestris* (Buff-tailed bumblebee) *(Apis terrestris)*
23	F1CGQ3	CLIP domain-containing serine protease (EC 3.4.21.-)	358		*Bombus terrestris* (Buff-tailed bumblebee) *(Apis terrestris)*
24	A0A232EG63	Complementary sex determination N-terminal domain-containing protein	399		*Trichomalopsis sarcophagae*
25	A0A7M7GSP5	Cysteine-rich venom protein 6 isoform X2	96		*Apis mellifera* (Honeybee)
26	A0A7M7GVB7	Cysteine-rich venom protein 1 isoform X1	99		*Apis mellifera* (Honeybee)
27	A0A7M7M0F6	Cysteine-rich venom protein	275		*Apis mellifera* (Honeybee)
28	A0A9B0BU67	Cysteine-rich venom protein 6	82		*Bombus terrestris* (Buff-tailed bumblebee) *(Apis terrestris)*
29	A0A9B0BUQ5	Cysteine-rich venom protein 6	91		*Bombus terrestris* (Buff-tailed bumblebee) *(Apis terrestris)*
30	B8Y9E0	C1q-like venom protein (C1q-like venom protein precursor)	163		*Apis mellifera* (Honeybee)
31	B8Y9E1	C1q-like venom protein	160		*Nasonia vitripennis* (Parasitic wasp)
32	V9IHG2	C1q-like venom protein	162		*Apis cerana* (Indian honeybee)
33	A0A2A3EM02	Dipeptidyl peptidase	778		*Apis cerana cerana* (Oriental honeybee)
34	Q08169	Hyaluronidase (Hya) (EC 3.2.1.35) (Allergen Api m II) (Hyaluronoglucosaminidase) (allergen Api m 2)	382	Hydrolyzes high molecular weight hyaluronic acid to produce small oligosaccharides.	*Apis mellifera* (Honeybee)
35	P0DQX4	Halictine-1 (HAL-1) (Halictin 1) (Halictine I)	12	Short linear cationic amphipathic alpha-helical antimicrobial peptide (AMP) with potent activity against both Gram-positive and Gram-negative bacteria, and	*Halictus sexcinctus* (Six-banded furrow bee) *(Apis sexcincta)*

				moderate activity against the yeast C.albicans. Has been tested on B.subtilis (MIC=0.8 uM), S.aureus (MIC=7.7 uM), E.coli (MIC=3.8 uM), P.aeruginosa (MIC=45 uM), and the yeast C.albicans (MIC=6.2 uM). Has also noticeable hemolytic activity (LC50=102 uM on human erythrocytes, and 82 uM on rat cells), indicating it cannot be considered for therapeutic application.<br>Interacts more strongly with anionic membranes compared to the zwitterionic ones because of the electrostatic contribution.<br>Interaction with anionic membranes is accompanied by structuring of the peptide as an alpha-helix and deep insertion into the membrane causing substantial membrane permeabilization at very low peptide/lipid molar ratios.<br>In the context of inflammation and cancer tests, is weakly cytotoxic to normal cells, induces calcium signaling but does not impact cAMP production.<br>In addition, prevents LPS-induced nitric oxid (NO) synthesis but does not affect the IP3 signaling and pro-inflammatory activation of endothelial cells .<br>Is cytotoxic towards cancer cells (HeLa S3 (IC50=11 uM), CRC SW 480 (IC50=44 uM), CCRF-CEM T (IC50=49 uM)), but does not show significant antiproliferative activity on the breast cancer cell line MDA-MB-231.	
36	P0DQX5	Halictine-2 (HAL-2) (Halictin 2) (Halictine II)	12	Short linear cationic amphipathic alpha-helical antimicrobial peptide (AMP) with potent activity against both Gram-positive and Gram-negative bacteria, and moderate activity against the yeast C.albicans. Has been	*Halictus sexcinctus* (Six-banded furrow bee) *(Apis sexcincta)*

				tested on B.subtilis (MIC=0.8 uM), S.aureus (MIC=7.7 uM), E.coli (MIC=3.8 uM), P.aeruginosa (MIC=45 uM), and the yeast C.albicans (MIC=6.2 uM). Has also noticeable hemolytic activity (LC50=102 uM on human erythrocytes, and 82 uM on rat cells), indicating it cannot be considered for therapeutic application .<br>Interacts more strongly with anionic membranes compared to the zwitterionic ones because of the electrostatic contribution.<br>Interaction with anionic membranes is accompanied by structuring of the peptide as an alpha-helix and deep insertion into the membrane causing substantial membrane permeabilization at very low peptide/lipid molar ratios.<br>In the context of inflammation and cancer tests, is weakly cytotoxic to normal cells, induces calcium signaling but does not impact cAMP production.<br>In addition, prevents LPS-induced nitric oxid (NO) synthesis but does not affect the IP3 signaling and pro-inflammatory activation of endothelial cells .<br>Is cytotoxic towards cancer cells (HeLa S3 (IC50=11 uM), CRC SW 480 (IC50=44 uM), CCRF-CEM T (IC50=49 uM)), but does not show significant antiproliferative activity on the breast cancer cell line MDA-MB-231.	
37	A0A2A3EHG0	Hyaluronidase (EC 3.2.1.35)	387		*Apis cerana cerana* (Oriental honeybee)
38	A0A7M7MTB6	Hyaluronidase (EC 3.2.1.35)	736		*Apis mellifera* (Honeybee)
39	A0A9B2JVW1	Hyaluronidase (EC 3.2.1.35)	381		*Bombus terrestris* (Buff-tailed bumblebee) *(Apis terrestris)*

40	A0A9C6SKU5	Hyaluronidase (EC 3.2.1.35)	336		*Bombus terrestris* (Buff-tailed bumblebee) *(Apis terrestris)*
41	I1VC83	Hyaluronidase (EC 3.2.1.35)	381		*Apis mellifera carnica* (Carniolan honeybee)
42	Q95PD7	Hyaluronidase (EC 3.2.1.35)	387		*Apis cerana cerana* (Oriental honeybee)
43	Q5EF78	Icarapin (Venom protein 2) (allergen Api m 10)	223		*Apis mellifera carnica* (Carniolan honeybee)
44	Q5BLY4	Icarapin-like (Venom carbohydrate-rich protein)	223		*Apis mellifera* (Honeybee)
45	A0A096XH32	Icarapin variant 3	151		*Apis mellifera carnica* (Carniolan honeybee)
46	A0A096XH33	Icarapin variant 4	87		*Apis mellifera carnica* (Carniolan honeybee)
47	A0A096XH34	Icarapin variant 5	59		*Apis mellifera carnica* (Carniolan honeybee)
48	A0A096XH35	Icarapin variant 2	200		*Apis mellifera carnica* (Carniolan honeybee)
49	A0A096XH37	Icarapin variant 8	41		*Apis mellifera carnica* (Carniolan honeybee)
50	A0A096XH38	Icarapin variant 9	25		*Apis mellifera carnica* (Carniolan honeybee)
51	A0A096XH39	Icarapin variant 10	19		*Apis mellifera carnica* (Carniolan honeybee)
52	A0A096XH40	Icarapin variant 7	45		*Apis mellifera carnica* (Carniolan honeybee)
53	A0A096XH51	Icarapin variant 1	204		*Apis mellifera carnica* (Carniolan honeybee)
54	A0A096XH57	Icarapin variant 6	47		*Apis mellifera carnica* (Carniolan honeybee)

55	A0A096XH63	Icarapin variant 11	12		*Apis mellifera carnica* (Carniolan honeybee)
56	A0A7M6UUY1	Icarapin-like precursor	223		*Apis mellifera* (Honeybee)
57	F2YPF6	Icarapin (Venom protein 2)	222		*Apis cerana* (Indian honeybee)
58	V9IFQ3	Inhibitor cysteine knot peptide	74		*Apis cerana* (Indian honeybee)
59	D8KY58	Kunitz-type serine protease inhibitor Bt-KTI	82	Serine protease inhibitor that inhibits plasmin (Ki=2.01 nM) and trypsin. Acts as an antifibrinolytic agent.	*Bombus terrestris* (Buff-tailed bumblebee) *(Apis terrestris)*
60	U3N283	Kazal-type serine protease inhibitor	128		*Apis cerana* (Indian honeybee)
61	P01501	Melittin (MEL) (MLT) (Allergen Api m 3) (Allergen Api m III) (allergen Api m 4)	70	Melittin: Main toxin of bee venom with strong antimicrobial activity and hemolytic activity; It has enhancing effects on bee venom phospholipase A2 activity; This amphipathic toxin binds to negatively charged membrane surface and forms pore by inserting into lipid bilayers inducing the leakage of ions and molecules and the enhancement of permeability that ultimately leads to cell lysis; It acts as a voltage-gated pore with higher selectivity for anions over cations; The ion conductance has been shown to be voltage-dependent; Self-association of melittin in membranes is promoted by high ionic strength, but not by the presence of negatively charged lipids; In vivo, intradermal injection into healthy human volunteers produce sharp pain sensation and an inflammatory response; It produces pain by activating primary nociceptor cells directly and indirectly due to its ability to activate plasma membrane phospholipase A2 and its pore-forming activity; In the context of inflammation and cancer tests, is highly cytotoxic to normal cells, highly induces calcium signaling and almost completely prevents cAMP production; In addition,	*Apis mellifera* (Honeybee)

				prevents LPS-induced nitric oxid (NO) synthesis but does not affect the IP3 signaling and pro-inflammatory activation of endothelial cells; Also shows significant antiproliferative activity on the breast cancer cell line MDA-MB-231.	
62	P01499	Mast cell degranulating peptide (MCD peptide) (MCDP) (Peptide 401)	50	Potent anti-inflammatory agent. At low concentrations, mediates the degranulation of mast cells thus evoking an inflammatory response. Also acts as a neurotoxin capable of blocking a class of voltage-gated potassium channels.	*Apis mellifera* (Honeybee)
63	Q8LW54	Melittin (MEL) (MLT)	77	Main toxin of bee venom with strong hemolytic activity and antimicrobial activity. It has enhancing effects on bee venom phospholipase A2 activity. This amphipathic toxin binds to negatively charged membrane surface and forms pore by inserting into lipid bilayers inducing the leakage of ions and molecules and the enhancement of permeability that ultimately leads to cell lysis. It acts as a voltage-gated pore with higher selectivity for anions over cations. The ion conductance has been shown to be voltage-dependent. Self-association of melittin in membranes is promoted by high ionic strength, but not by the presence of negatively charged lipids. In vivo, intradermal injection into healthy human volunteers produce sharp pain sensation and an inflammatory response. It produces pain by activating primary nociceptor cells directly and indirectly due to its ability to activate plasma membrane phospholipase A2 and its pore-forming activity.	*Apis cerana* (Indian honeybee)
64	P01502	Melittin (MEL) (MLT)	26	Main toxin of bee venom with strong hemolytic activity and antimicrobial activity. It has enhancing effects on bee venom phospholipase A2 activity. This amphipathic toxin	*Apis dorsata* (Giant honeybee)

				binds to negatively charged membrane surface and forms pore by inserting into lipid bilayers inducing the leakage of ions and molecules and the enhancement of permeability that ultimately leads to cell lysis. It acts as a voltage-gated pore with higher selectivity for anions over cations. The ion conductance has been shown to be voltage-dependent. Self-association of melittin in membranes is promoted by high ionic strength, but not by the presence of negatively charged lipids. In vivo, intradermal injection into healthy human volunteers produce sharp pain sensation and an inflammatory response. It produces pain by activating primary nociceptor cells directly and indirectly due to its ability to activate plasma membrane phospholipase A2 and its pore-forming activity.	
65	P01504	Melittin (MEL) (MLT)	26	Main toxin of bee venom with strong hemolytic activity and antimicrobial activity. It has enhancing effects on bee venom phospholipase A2 activity. This amphipathic toxin binds to negatively charged membrane surface and forms pore by inserting into lipid bilayers inducing the leakage of ions and molecules and the enhancement of permeability that ultimately leads to cell lysis. It acts as a voltage-gated pore with higher selectivity for anions over cations. The ion conductance has been shown to be voltage-dependent. Self-association of melittin in membranes is promoted by high ionic strength, but not by the presence of negatively charged lipids. In vivo, intradermal injection into healthy human volunteers produce sharp pain sensation and an inflammatory response. It produces pain by activating primary	*Apis florea* (Dwarf honeybee)

				nociceptor cells directly and indirectly due to its ability to activate plasma membrane phospholipase A2 and its pore-forming activity.	
66	P0DPR9	Melittin-N (MEL-N) (MLT)	70	Main toxin of bee venom with strong hemolytic activity and antimicrobial activity. It has enhancing effects on bee venom phospholipase A2 activity; Shows lower cytotoxicity when tested on E.coli and cancer cell lines than melittin, as well as lower anti-inflammatory properties and lower properties to interact to small unilamellar liposomes.	*Apis cerana* (Indian honeybee)
67	P68407	Melittin (MEL) (MLT)	70	Main toxin of bee venom with strong hemolytic activity and antimicrobial activity. It has enhancing effects on bee venom phospholipase A2 activity. This amphipathic toxin binds to negatively charged membrane surface and forms pore by inserting into lipid bilayers inducing the leakage of ions and molecules and the enhancement of permeability that ultimately leads to cell lysis. It acts as a voltage-gated pore with higher selectivity for anions over cations. The ion conductance has been shown to be voltage-dependent. Self-association of melittin in membranes is promoted by high ionic strength, but not by the presence of negatively charged lipids. In vivo, intradermal injection into healthy human volunteers produce sharp pain sensation and an inflammatory response. It produces pain by activating primary nociceptor cells directly and indirectly due to its ability to activate plasma membrane phospholipase A2 and its pore-forming activity.	*Apis cerana* (Oriental honeybee)

68	P04567	Mast cell degranulating peptide (MCD peptide) (MCDP)	28	Mast cell degranulating peptide.	*Bombus pensylvanicus* (American bumblebee) *(Apis pensylvanica)*
69	Q6H2Z4	Mast cell degranulating peptide	50	Potent anti-inflammatory agent. At low concentrations, mediates the degranulation of mast cells thus evoking an inflammatory response. Also acts as a neurotoxin capable of blocking a class of voltage-gated potassium channels.	*Apis cerana* (Oriental honeybee)
70	P00630	Phospholipase A2 (bvPLA2) (EC 3.1.1.4) (Allergen Api m I) (Phosphatidylcholine 2-acylhydrolase) (allergen Api m 1)	167	PLA2 catalyzes the calcium-dependent hydrolysis of the 2-acyl groups in 3-sn-phosphoglycerides.	*Apis mellifera* (Honeybee)
71	P82971	Phospholipase A2 (PLA2) (EC 3.1.1.4) (Phosphatidylcholine 2-acylhydrolase) (allergen Bom t 1)	136	PLA2 catalyzes the calcium-dependent hydrolysis of the 2-acyl groups in 3-sn-phosphoglycerides.	*Bombus terrestris* (Buff-tailed bumblebee) *(Apis terrestris)*
72	Q7M4I5	Phospholipase A2 (PLA2) (EC 3.1.1.4) (Phosphatidylcholine 2-acylhydrolase) (allergen Api d 1)	134	PLA2 catalyzes the calcium-dependent hydrolysis of the 2-acyl groups in 3-sn-phosphoglycerides.	*Apis dorsata* (Giant honeybee)
73	Q7M4I6	Phospholipase A2 (PLA2) (EC 3.1.1.4) (Phosphatidylcholine 2-acylhydrolase) (allergen Bom p 1)	136	PLA2 catalyzes the calcium-dependent hydrolysis of the 2-acyl groups in 3-sn-phosphoglycerides.	*Bombus pensylvanicus* (American bumblebee) *(Apis pensylvanica)*
74	Q9BMK4	Phospholipase A2 (PLA2) (EC 3.1.1.4) (Phosphatidylcholine 2-acylhydrolase) (allergen Api c 1)	134	PLA2 catalyzes the calcium-dependent hydrolysis of the 2-acyl groups in 3-sn-phosphoglycerides.	*Apis cerana cerana* (Oriental honeybee)
75	A0A0N6XWN3	PVF1 variant 2	272		*Apis mellifera carnica* (Carniolan honeybee)
76	A0A0N6XWQ5	PVF1 variant 3	249		*Apis mellifera carnica* (Carniolan honeybee)
77	A0A0N6XWR9	PVF1 variant 1	292		*Apis mellifera carnica* (Carniolan honeybee)

78	B7UUK1	phospholipase A2 (EC 3.1.1.4)	167	PLA2 catalyzes the calcium-dependent hydrolysis of the 2-acyl groups in 3-sn-phosphoglycerides.	*Apis mellifera* (Honeybee)
79	A0A0K1YW63	Secapin (AcSecapin-1)	115	Serine protease inhibitor which exhibits antifibrinolytic, antielastolytic and antimicrobial activities .<br>Displays antimicrobial activity against bacteria and fungi. Likely functions in the innate immune response to microbial infection and possibly in the venom, as an antifibrinolytic agent.<br>The recombinant form inhibits trypsin (IC50=80.02 nM, Ki=127.25 nM), chymotrypsin (IC50=393.78 nM, Ki=432.59 nM), the microbial serine proteases subtilisin A (IC50=379.20 nM, Ki=492.77 nM) and proteinase K (IC50=189.43 nM, Ki=271.76 nM), plasmin (IC50=457.98 nM, Ki=502.91 nM), human elastase (IC50=347.81 nM, Ki=469.90 nM) and porcine elastase (IC50=94.70 nM, Ki=125.62 nM).<br>Does not inhibit thrombin.<br>Binds to human plasmin and inhibits the plasmin-mediated degradation of fibrin to fibrin degradation products.<br>Also binds to bacterial and fungal surfaces and exhibits antimicrobial activity against the Gram-positive bacteria B.thuringiensis (MIC=4.21 uM) and P.larvae (MIC=11.13 uM), the Gram-negative bacterium E.coli (MIC=6.50 uM), and the fungus B.bassiana (IC50=2.57 uM).<br>The synthetic peptide also exhibits antimicrobial activity against the Gram-positive bacterium P.larvae (MIC=41.12 uM), the Gram-negative bacterium P.aeruginosa (MIC=65.75 uM), and the fungus B.bassiana (IC50=44.27 uM) (Ref.2). In vitro it does not induce an	*Apis cerana* (Indian honeybee)

				inflammatory response and has no cytotoxic activity against mouse embryo cells.	
80	I1VC85	Secapin-2	77	Serine protease inhibitor which exhibits antifibrinolytic, antielastolytic and antimicrobial activities (By similarity); Displays antimicrobial activity against bacteria and fungi (By similarity); Likely functions in the innate immune response to microbial infection and possibly in the venom, as an antifibrinolytic agent (By similarity); Induces hyperalgesia and edema mediated by leukotrienes when injected into mice; Does not induce hemolytic activity, mast cell degranulation, or chemotactic activity for polymorphonucleated leukocytes (PMNL).	*Apis mellifera* (Honeybee)
81	P02852	Secapin	77		*Apis mellifera* (Honeybee)
82	C0HLU0	Secapin-1	25	Serine protease inhibitor which exhibits antifibrinolytic, antielastolytic and antimicrobial activities (By similarity); Displays antimicrobial activity against bacteria and fungi (By similarity); Likely functions in the innate immune response to microbial infection and possibly in the venom, as an antifibrinolytic agent (By similarity).	*Apis mellifera* (Honeybee)
83	A0A1B1JIC6	Superoxide dismutase [Cu-Zn] (EC 1.15.1.1)	168	Destroys radicals which are normally produced within the cells and which are toxic to biological systems.	*Bombus terrestris* (Buff-tailed bumblebee) *(Apis terrestris)*
84	A0A1B1JID1	Superoxide dismutase [Cu-Zn] (EC 1.15.1.1)	177	Destroys radicals which are normally produced within the cells and which are toxic to biological systems.	*Apis mellifera* (Honeybee)
85	A0A1B1JID3	Superoxide dismutase [Cu-Zn] (EC 1.15.1.1)	183	Destroys radicals which are normally produced within the cells and which are toxic to biological systems.	*Apis cerana* (Indian honeybee)
86	A0A2A3ERB7	Snake venom vascular endothelial growth factor toxin	153		*Apis cerana* (Oriental honeybee)
87	Q7YWB0	Secapin	77	Nontoxic peptide.	*Apis cerana* (Oriental honeybee)
88	A0A8J2H6X6	Similar to Venom serine carboxypeptidase (Apis mellifera)	337		*Cotesia congregata* (Parasitoid wasp) *(Apanteles congregatus)*

89	A0A8J2HEU3	Similar to Venom acid phosphatase Acph-1 (Apis mellifera)	398		*Cotesia congregata* (Parasitoid wasp) *(Apanteles congregatus)*
90	A0A8J2HFP8	Similar to Venom dipeptidyl peptidase 4 (Apis mellifera)	635		*Cotesia congregata* (Parasitoid wasp) *(Apanteles congregatus)*
91	A0A8J2HN43	Similar to Venom acid phosphatase Acph-1 (Apis mellifera)	385		*Cotesia congregata* (Parasitoid wasp) *(Apanteles congregatus)*
92	A0A8J2HPI4	Similar to Venom acid phosphatase Acph-1 (Apis mellifera)	272		*Cotesia congregata* (Parasitoid wasp) *(Apanteles congregatus)*
93	A0A8J2HQ83	Similar to Venom acid phosphatase Acph-1 (Apis mellifera)	353		*Cotesia congregata* (Parasitoid wasp) *(Apanteles congregatus)*
94	A0A8J2HT38	Similar to Venom acid phosphatase Acph-1 (Apis mellifera)	409		*Cotesia congregata* (Parasitoid wasp) *(Apanteles congregatus)*
95	P56587	Tertiapin (TPN)	21	Presynaptic neurotoxin that blocks the inwardly rectifying Kir1.1/KCNJ1 and Kir3.1/3.4 (KCNJ3/KCNJ5) potassium channels with high affinity by binding to the M1-M2 linker region of these channels in a 1:1 stoichiometry. It may block the potassium channel pore by occluding its alpha helix into the channel vestibule. Tertiapin-Q also inhibits calcium-activated large conductance BK-type (KCNMA) potassium channels in a concentration-, and voltage-dependent manner, in addition to inhibiting Kir3.1/3.2 (KCNJ3/KCNJ6) heteromultimers potassium channels. It can prevent dose-dependently acetylcholine(ACh)-induced atrioventricular blocks in mammalian hearts, as KCNJ3/KCNJ5 channels	*Apis mellifera* (Honeybee)

				(also named I(KACh), because these channels are activated by ACh) are found in mammalian myocytes. Interacts specifically with calmodulin in the presence of calcium.	
96	B2D0J4	Venom dipeptidyl peptidase 4 (Allergen C) (Venom dipeptidyl peptidase IV) (EC 3.4.14.5) (allergen Api m 5)	775	Venom dipeptidyl-peptidase which removes N-terminal dipeptides sequentially from polypeptides having unsubstituted N-termini provided that the penultimate residue is proline. May process promelittin into its active form and/or modulate the chemotactic activity of immune cells after the insect sting.	*Apis mellifera* (Honeybee)
97	A0A2R4SV19	Venom serine protease inhibitor (AcVSPI) (VSPI) (Allergen Api m 6-like peptide)	87	Antifibrinolytic and antimicrobial serine protease inhibitor. Inhibits trypsin, plasmin and microbial serine proteases but not chymotrypsin, thrombin and elastase. Inhibits the plasmin-mediated degradation of fibrin to fibrin degradation products. Also binds to bacterial and fungal surfaces and exhibits antimicrobial activity against fungi as well as Gram-positive and Gram-negative bacteria.	*Apis cerana* (Indian honeybee)
98	B2D0J5	Venom carboxylesterase-6 (EC 3.1.1.1) (allergen Api m 8)	557		*Apis mellifera* (Honeybee)
99	Q5BLY5	Venom acid phosphatase Acph-1 (EC 3.1.3.2) (allergen Api m 3)	388		*Apis mellifera* (Honeybee)
100	Q8MQS8	Venom serine protease 34 (SP34) (EC 3.4.21.-) (allergen Api m 7)	405		*Apis mellifera* (Honeybee)
101	A0A3G2LYW8	Vitellogenin	1770		*Apis cerana* (Indian honeybee)
102	A0A7M7FZB3	Venom acid phosphatase Acph-1	361		*Apis mellifera* (Honeybee)
103	A0A7M7G121	Venom acid phosphatase Acph-1-like	410		*Apis mellifera* (Honeybee)
104	A0A7M7G2H3	Venom acid phosphatase Acph-1	391		*Apis mellifera* (Honeybee)
105	A0A7M7GY01	Venom acid phosphatase Acph-1	361		*Apis mellifera* (Honeybee)

106	A0A7M7GZ22	Venom acid phosphatase Acph-1	410		*Apis mellifera* (Honeybee)
107	A0A7M7H3H4	Venom acid phosphatase Acph-1	361		*Apis mellifera* (Honeybee)
108	A0A7M7IKA6	Venom acid phosphatase Acph-1 isoform X1	410		*Apis mellifera* (Honeybee)
109	A0A7M7L7I4	Venom acid phosphatase Acph-1	361		*Apis mellifera* (Honeybee)
110	A0A7M7MWH5	Venom metalloproteinase 3 isoform X4	590		*Apis mellifera* (Honeybee)
111	A0A9B0BUL5	Venom acid phosphatase Acph-1	385		*Bombus terrestris* (Buff-tailed bumblebee) *(Apis terrestris)*
112	A0A9B0BW32	Venom acid phosphatase Acph-1	360		*Bombus terrestris* (Buff-tailed bumblebee) *(Apis terrestris)*
113	A0A9B0C0T5	Venom acid phosphatase Acph-1	415		*Bombus terrestris* (Buff-tailed bumblebee) *(Apis terrestris)*
114	A0A9B2JRG7	Venom acid phosphatase Acph-1 isoform X1	384		*Bombus terrestris* (Buff-tailed bumblebee*) (Apis terrestris)*
115	A0A9C6SPZ5	Venom acid phosphatase Acph-1	383		*Bombus terrestris* (Buff-tailed bumblebee) *(Apis terrestris)*
116	A0A9C6W6L5	Venom acid phosphatase Acph-1 isoform X1	407		*Bombus terrestris* (Buff-tailed bumblebee) *(Apis terrestris)*
117	C9WMM5	Venom serine carboxypeptidase (EC 3.4.16.5) (allergen Api m 9)	467		*Apis mellifera* (Honeybee)
118	G8IIT0	Vitellogenin	1756		*Vespula vulgaris* (Yellow jacket) (Wasp)
119	P0CH88	Venom protease (EC 3.4.21.-) (allergen Bom t 4)	20		*Bombus terrestris* (Buff-tailed bumblebee) *(Apis terrestris)*
120	Q7M4I3	Venom protease (EC 3.4.21.-) (allergen Bom p 4)	243		*Bombus pensylvanicus* (American bumblebee) *(Apis pensylvanica)*
121	A0A060GL20	Venom acid phosphatase	401		*Apis cerana* (Indian honeybee*)*

122	A0A060GPB1	Venom acid phosphatase Acph-1-like protein	408		*Apis cerana* (Indian honeybee)
123	A0A087ZYX8	Venom serine protease 34 isoform X1	405		*Apis mellifera* (Honeybee)
124	A0A2A3EG95	Venom allergen	195		*Apis cerana cerana* (Oriental honeybee)
125	A0A2A3EKS9	Venom allergen	297		*Apis cerana cerana* (Oriental honeybee)
126	A0A6P5I706	Venom dipeptidyl peptidase 4 isoform X2	795		*Bombus terrestris* (Buff-tailed bumblebee) *(Apis terrestris)*
127	A0A7M6UUV6	Venom dipeptidyl peptidase 4	779		*Apis mellifera* (Honeybee)
128	A0A7M7GLK1	Venom dipeptidyl peptidase 4-like isoform X1	830		*Apis mellifera* (Honeybee)
129	A0A7M7GMA2	Venom dipeptidyl peptidase 4-like isoform X2	796		*Apis mellifera* (Honeybee)
130	A0A7M7GMX5	Venom dipeptidyl peptidase 4	795		*Apis mellifera* (Honeybee)
131	A0A7M7GUP2	Venom acid phosphatase Acph-1	391		*Apis mellifera* (Honeybee)
132	A0A7M7GWB1	Venom carboxylesterase-6-like	651		*Apis mellifera* (Honeybee)
133	A0A7M7IFA3	Venom allergen 3	137		*Apis mellifera* (Honeybee)
134	A0A7M7IHL1	Venom acid phosphatase Acph-1 isoform X1	401		*Apis mellifera* (Honeybee)
135	A0A7M7IJ45	Venom peptide isomerase heavy chain	299		*Apis mellifera* (Honeybee)
136	A0A7M7L3N3	Venom serine protease 34 isoform X2	403		*Apis mellifera* (Honeybee)
137	A0A7M7M4B2	Venom acid phosphatase Acph-1 isoform X2	346		*Apis mellifera* (Honeybee)
138	A0A7M7MQD1	Venom dipeptidyl peptidase 4 isoform X1	775		*Apis mellifera* (Honeybee)

139	A0A7M7MSN5	Venom serine protease 34 isoform X1	375		*Apis mellifera* (Honeybee)
140	A0A7M7R4Y2	Venom dipeptidyl peptidase 4-like isoform X3	795		*Apis mellifera* (Honeybee)
141	A0A8U1GQ41	Venom serine protease 34 (EC 3.4.21.-)	405		*Apis mellifera* (Honeybee)
142	A0A9B0BJI5	Venom dipeptidyl peptidase 4 isoform X1	778		*Bombus terrestris* (Buff-tailed bumblebee) *(Apis terrestris)*
143	A0A9B0BRA6	Venom serine protease 34	405		*Bombus terrestris* (Buff-tailed bumblebee) *(Apis terrestris)*
144	A0A9B0F5F9	Venom acid phosphatase Acph-1 isoform X2	370		*Bombus terrestris* (Buff-tailed bumblebee) *(Apis terrestris)*
145	A0A9B2JP51	Venom dipeptidyl peptidase 4 isoform X1	796		*Bombus terrestris* (Buff-tailed bumblebee) *(Apis terrestris)*
146	A0A9B2MN63	Venom protease isoform X1	298		*Bombus terrestris* (Buff-tailed bumblebee) *(Apis terrestris)*
147	A0A9B2MQ39	Venom allergen 5.02	237		*Bombus terrestris* (Buff-tailed bumblebee) *(Apis terrestris)*
148	A0A9B2MSN2	Venom acid phosphatase Acph-1 isoform X1	424		*Bombus terrestris* (Buff-tailed bumblebee) *(Apis terrestris)*
149	A0A9B7HZX4	Venom dipeptidyl peptidase 4 isoform X2	672		*Bombus terrestris* (Buff-tailed bumblebee) *(Apis terrestris)*
150	A0A9C6S3I2	Venom acid phosphatase Acph-1 isoform X3	408		*Bombus terrestris* (Buff-tailed bumblebee) *(Apis terrestris)*
151	A0A9C6SAZ5	Venom allergen 3-like	309		*Bombus terrestris* (Buff-tailed bumblebee) *(Apis terrestris)*
152	A0A9C6SH96	Venom acid phosphatase Acph-1 isoform X2	411		*Bombus terrestris* (Buff-tailed bumblebee) *(Apis terrestris)*
153	A0A9C6SI46	Venom protease-like	301		*Bombus terrestris* (Buff-tailed bumblebee) *(Apis terrestris)*

154	A0A9C6SP64	Venom dipeptidyl peptidase 4 isoform X3	784		*Bombus terrestris* (Buff-tailed bumblebee) *(Apis terrestris)*
155	A0A9C6VVC6	Venom serine protease Bi-VSP isoform X3	236		*Bombus terrestris* (Buff-tailed bumblebee) *(Apis terrestris)*
156	A0A9C6VYF6	Venom protease isoform X2	295		*Bombus terrestris* (Buff-tailed bumblebee) *(Apis terrestris)*
157	A0A9C6W3W7	Venom acid phosphatase Acph-1 isoform X2	361		*Bombus terrestris* (Buff-tailed bumblebee) *(Apis terrestris)*
158	A0A9C6W4A7	Venom protease-like	249		*Bombus terrestris* (Buff-tailed bumblebee) *(Apis terrestris)*
159	A5HNY6	Venom protease	182		*Bombus terrestris* (Buff-tailed bumblebee) *(Apis terrestris)*
160	F1CGQ4	Venom bombolitin 1	56		*Bombus terrestris* (Buff-tailed bumblebee) *(Apis terrestris)*
161	F1CGQ5	Venom bombolitin 2	56		*Bombus terrestris* (Buff-tailed bumblebee) *(Apis terrestris)*
162	A0A7M7H308	Waprin-Thr1	110	Antimicrobial peptides with activity against Gram-positive and Gram-negative bacteria as well as fungi; Recognizes carbohydrates in the microbial cell walls, and induces structural damage to them; Also inhibits microbial serine proteases subtilisin A and proteinase K, as well as human and porcine elastases; Carbohydrates that are recognized are LPS, mannan, peptidoglycan, and N-acetl-D-glucosamine.	*Apis mellifera* (Honeybee)

Table S2. The Summary Table of Wasp (*Vespa*) Venom Protein and Peptide Constituents.

	Entry	Protein names	Length	Pharmacological effect	Organism
1	B7SD94	Anticoagulant serine protease	305		*Vespa magnifica* (Hornet)

2	P01518	Crabrolin	13	Antimicrobial and mast cell degranulating peptide.<br>Shows low antimicrobial activity towards some Gram-negative bacteria (E.coli MIC=300 µg/mL, S.typhimurium MIC=300 µg/mL) and all Gram-positive bacteria tested (B.subtilis MIC=300 µg/mL, S.aureus MIC=300 µg/mL, E.faecalis MIC=150 µg/mL, and E.faecium MIC=75 µg/mL).<br>May act by permeabilizing membranes, since it permeabilizes liposomes composed by phosphatidylethanolamine (PE) / phosphatidylglycerol (PG) and cardiolipin (CL) / phosphatidylglycerol (PG), which mimic the membranes of Gram-negative and Gram-positive bacteria, respectively.<br>Causes hemolysis on rat and guinea pig erythrocytes.<br>Its mast cell degranulation activity may be related to the activation of G-protein coupled receptors in mast cells as well as interaction with other proteins located in cell endosomal membranes in the mast cells (By similarity).	*Vespa crabro* (European hornet)
3	P0C1M6	Dominulin-A	17	Shows antimicrobial activity against the Gram-positive bacteria B.subtilis ATCC 6633 (MIC=2 µg/mL), and the Gram-negative bacteria E.coli JM109 (MIC=8 µg/mL).	*Polistes dominula* (European paper wasp) *(Vespa dominula)*
4	P0C1M7	Dominulin-B	17	Shows antimicrobial activity against the Gram-positive bacteria B.subtilis ATCC 6633 (MIC=2 µg/mL), and the Gram-negative bacteria E.coli JM109 (MIC=8 µg/mL).	*Polistes dominula* (European paper wasp) *(Vespa dominula)*
5	A4UA14	Dipeptidylpeptidase IV (EC 3.4.14.5)	775		*Vespa basalis* (Hornet)
6	D1MEJ4	DTX protein	78		*Eumenes pomiformis* (Potter wasp) *(Vespa pomiformis)*
7	D1MEI6	Eumenitin VP1 (Venom peptide 1) (EpVP1) (VP1)	63	Antimicrobial peptide with activities against the fungi B.cinerea (MIC=5 uM) and C.albicans (MIC=100 uM),	*Eumenes pomiformis* (Potter wasp) *(Vespa pomiformis)*

				the Gram-negative bacterium E.coli (MIC=25 uM) and the Gram-positive bacterium S.aureus (MIC=100 uM).<br>Shows cytolytic activity against insect cell lines.<br>Has no hemolytic activity against human erythrocytes.<br>In vivo, peptide injection in the vicinity of the head and thorax of lepidopteran larvae induces feeding disorder followed by death due to starvation.	
8	D1MEJ6	Hyaluronidase (EC 3.2.1.35)	372		*Eumenes pomiformis* (Potter wasp) *(Vespa pomiformis)*
9	D1MEJ8	Histidine decarboxylase (EC 4.1.1.22)	502		*Eumenes pomiformis* (Potter wasp) *(Vespa pomiformis)*
10	C0HLL4	Hyaluronidase A (EC 3.2.1.35) (Vesp v 2A)	341		*Vespa velutina* (Asian yellow-legged hornet)
11	C0HLL5	Hyaluronidase B (EC 3.2.1.35) (Vesp v 2B)	331		*Vespa velutina* (Asian yellow-legged hornet)
12	P86875	Hyaluronidase (Hya) (EC 3.2.1.35) (Hyaluronoglucosaminidase) (allergen Vesp ma 2)	357	Hydrolyzes high molecular weight hyaluronic acid to produce small oligosaccharides	*Vespa magnifica* (Hornet)
13	P17236	Histamine-releasing peptide 2 (HR-2) (HR2) (Histamine-releasing peptide II) (HR-II) (HRII) (Mast cell degranulating peptide HR2) (MCD HR2)	14	Mast cell degranulating peptide. Induces the chemotaxis of neutrophils. Its mast cell degranulation activity may be related to the activation of G-protein coupled receptors in mast cells as well as interaction with other proteins located in cell endosomal membranes in the mast cells (By similarity).	*Vespa orientalis* (Oriental hornet)
14	A0A9E7V465	Hyaluronidase (EC 3.2.1.35)	356		*Vespa tropica* (Greater banded hornet) (Sphex tropica)
15	C0LNR2	Kunitz-type serine protease inhibitor bicolin	77	Serine protease inhibitor that inhibits trypsin (Ki=550 nM) and thrombin (Ki=26000 nM). Exerts anticoagulant activity probably by the way of inhibiting thrombin.	*Vespa bicolor* (Black shield wasp)

16	A0SPI0	Mastoparan-like peptide 12c	40	Shows mast cell degranulation and antimicrobial activities against the Gram-negative bacteria E.coli ATCC 25922 (MIC=6.0 μg/mL), the Gram-positive bacteria S.aureus ATCC 2592 (MIC=3.0 μg/mL) and the fungus C.albicans ATCC 2002 (MIC=12 μg/mL); Exhibits little hemolytic activity against washed human erythrocytes (By similarity). Its mast cell degranulation activity may be related to the activation of G-protein coupled receptors in mast cells as well as interaction with other proteins located in cell endosomal membranes in the mast cells (By similarity).	*Vespa magnifica* (Hornet)
17	P04205	Mastoparan-M (MP-M) (Mast cell-degranulating peptide)	60	Antimicrobial and mast cell degranulating peptide; Has broad spectrum antibacterial activity against both Gram-positive (S.aureus MIC=32-64 μg/mL, S.xylosus MIC=2 μg/mL, and S.alactolyticus MIC=16 μg/mL) and Gram-negative bacteria (C.koseri MIC=4 μg/mL, E.coli MIC=8 μg/mL, K.pneumoniae MIC=48 μg/mL, P.aerugiosa MIC=128 μg/mL, S.choleraesuis MIC=32 μg/mL, S.typhimurium MIC=32 μg/mL, and V.parahamelytics MIC=64 μg/mL); Affects membrane permeability of E.coli ;Shows hemolytic activities on sheep, chicken and human erythrocytes;Its mast cell degranulation activity may be related to the activation of G-protein coupled receptors in mast cells as well as interaction with other proteins located in cell endosomal membranes in the mast cells (By similarity); In the mouse macrophage cells, stimulates the production of TNF-alpha, IL-1beta, and nitrite (which reflect nitric oxide synthesis); mRNA levels of TNF-alpha are also increased indicating that this	*Vespa mandarinia* (Asian giant hornet)

				peptide is able to induce an immune response and the recruitment of defense cells.	
18	P0C1Q7	Mastoparan-VT (MpVT) (Mastoparan-T)	37	Antimicrobial peptide with potent activity against both Gram-positive (S.aureus MIC=50 µg/mL, and B.subtilis MIC=25 µg/mL) and Gram-negative bacteria (P.aeruginosa MIC=25 µg/mL, E.coli MIC=3-50 µg/mL, K.pneumoniae MIC=25 µg/mL). Exhibits little hemolytic activity on human erythrocytes.	*Vespa tropica* (Greater banded hornet) (Sphex tropica)
19	P0DQZ4	Mastoparan-VT1 [Cleaved into: Mastoparan-VT2]	60	Mastoparan-VT1<br>Antimicrobial peptide with activities against Gram-negative and Gram-positive bacteria and the fungi C.albicans and C.parapsilosis; Exhibits little hemolytic activity against washed human erythrocytes; Also acts as a mast cell degranulating peptide (By similarity); Its mast cell degranulation activity may be related to the activation of G-protein coupled receptors in mast cells as well as interaction with other proteins located in cell endosomal membranes in the mast cells (By similarity). Mastoparan-VT2<br>Antimicrobial peptide with activities against Gram-negative and Gram-positive bacteria and the fungi C.albicans and C.parapsilosis; Exhibits little hemolytic activity against washed human erythrocytes; Also acts as a mast cell degranulating peptide (By similarity).	*Vespa tropica* (Greater banded hornet) (Sphex tropica)
20	P0DRA1	Mastoparan-VB1 (MP-VB1)	60	Antimicrobial peptide. Shows activity against both Gram-positive (S.aureus MIC=1.9-3.75 µg/mL) and -negative (E.coli MIC=15-60 µg/mL) bacteria, as well against fungi (C.albicans MIC=15 µg/mL). Also promotes moderate mast cell degranulation. Does not show hemolytic activity on rabbit and human erythrocytes.	*Vespa bicolor* (Black shield wasp)

				Its mast cell degranulation activity may be related to the activation of G-protein coupled receptors in mast cells as well as interaction with other proteins located in cell endosomal membranes in the mast cells (By similarity).	
21	P21654	Mastoparan-B (MP-B)	60	Antimicrobial and mast cell degranulating peptide. Has broad spectrum antibacterial activity against both Gram-positive (S.aureus MIC=96-128 μg/mL, S.xylosus MIC=2 μg/mL, S.alactolyticus MIC=32 μg/mL, and S.choleraesuis MIC=32 μg/mL) and Gram-negative bacteria (C.koseri MIC=6 μg/mL, E.coli MIC=3-16 μg/mL, K.pneumoniae MIC=128 μg/mL, P.aerugiosa MIC=128 μg/mL, S.typhimurium MIC=64 μg/mL, V.parahamelytics MIC=32 μg/mL, and S.enterica), as well as on fungi (C.albicans, C.glabrata, and C.neoformans).<br>Does not show antimicrobial activity against S.mutans.<br>Affects membrane permeability of E.coli.<br>Also acts as a mast cell degranulating peptide, that causes liberation of histamine from rat peritoneal mast cells.<br>Its mast cell degranulation activity may be related to the activation of G-protein coupled receptors in mast cells as well as interaction with other proteins located in cell endosomal membranes in the mast cells (By similarity).<br>Whether this peptide shows hemolytic activities is contreversial, as Lin et al., 2011 and Ho et al., 1991 found a hemolytic activity on sheep, chicken and human erythrocytes, whereas Kim et al., 2016 found no hemolytic activity on human erythrocytes.<br>In vivo, induces edema in the rat paw.	*Vespa basalis* (Hornet)

22	P01515	Mastoparan-X (MP-X) (MPX)	14	Antimicrobial peptide with moderate activity on Gram-positive (S.mutans, and S.aureus), and Gram-negative bacteria (S.enterica), as well as on fungi (C.albicans, C.glabrata, and C.neoformans).<br>Also acts as a mast cell degranulating peptide (PubMed:540363). Its mast cell degranulation activity may be related to the activation of G-protein coupled receptors in mast cells as well as interaction with other proteins located in cell endosomal membranes in the mast cells (By similarity).<br>Activates G proteins that couple to phospholipase C (Probable). Does not cause hemolysis to human erythrocytes.<br>Decreases the proliferation and viability of human leukemia HL60 cells.<br>Has a membranolytic activity on human glioblastoma multiforme cells (brain tumor cells) that leads to cell necrosis.	*Vespa xanthoptera* (Japanese yellow hornet) *(Vespa simillima xanthoptera)*
23	P01516	Mastoparan-C (MP-C)	14	Mast cell degranulating peptide.<br>Causes hemolysis of guinea pig erythrocytes.<br>Its mast cell degranulation activity may be related to the activation of G-protein coupled receptors in mast cells as well as interaction with other proteins located in cell endosomal membranes in the mast cells (By similarity).	*Vespa crabro* (European hornet)
24	P0C1M4	Mastoparan-like peptide 12a	14	Antimicrobial and mast cell degranulating peptide. Shows antimicrobial activity against the Gram-negative bacteria E.coli ATCC 25922 (MIC=7.5 μg/mL), the Gram-positive bacteria S.aureus ATCC 2592 (MIC=3.7 μg/mL) and the fungus C.albicans ATCC 2002 (MIC=3.7 μg/mL). Has little hemolytic activity; Its mast cell degranulation	*Vespa magnifica* (Hornet)

				activity may be related to the activation of G-protein coupled receptors in mast cells as well as interaction with other proteins located in cell endosomal membranes in the mast cells (By similarity).	
25	P0C1M5	Mastoparan-like peptide 12b	13	Antimicrobial and mast cell degranulating peptide. Shows antimicrobial activity against the Gram-negative bacteria E.coli ATCC 25922 (MIC=15 μg/mL), the Gram-positive bacteria S.aureus ATCC 2592 (MIC=3.7 μg/mL) and the fungus C.albicans ATCC 2002 (MIC=7.5 μg/mL). Has little hemolytic activity.<br>Its mast cell degranulation activity may be related to the activation of G-protein coupled receptors in mast cells as well as interaction with other proteins located in cell endosomal membranes in the mast cells (By similarity).	*Vespa magnifica* (Hornet)
26	P0C5G7	Mastoparan-like peptide 12d	14	Shows mast cell degranulation and antimicrobial activities against the Gram-negative bacteria E.coli ATCC 25922 (MIC=3.0 μg/mL), the Gram-positive bacteria S.aureus ATCC 2592 (MIC=1.5 μg/mL) and the fungus C.albicans ATCC 2002 (MIC=12.0 μg/mL); Its mast cell degranulation activity may be related to the activation of G-protein coupled receptors in mast cells as well as interaction with other proteins located in cell endosomal membranes in the mast cells (By similarity).	*Vespa magnifica* (Hornet)
27	P0C1Q6	Mastoparan-A (MP-A)	60	Antimicrobial and mast cell degranulating peptide. Has broad spectrum antibacterial activity against both Gram-positive and Gram-negative bacteria (S.aureus MIC=32-64 μg/mL, S.xylosus MIC=2 μg/mL, S.alactolyticus MIC=12 μg/mL, C.koseri MIC=4 μg/mL, E.coli MIC=8 μg/mL, K.pneumoniae MIC=32 μg/mL, P.aerugiosa MIC=192 μg/mL, S.choleraesuis MIC=32 μg/mL,	*Vespa analis* (Yellow-vented hornet)

				S.typhimurium MIC=32 μg/mL, V.parahamelytics MIC=16 μg/mL). Affects membrane permeability of E.coli. Shows hemolytic activities on sheep, chicken and human erythrocytes.<br>Its mast cell degranulation activity may be related to the activation of G-protein coupled receptors in mast cells as well as interaction with other proteins located in cell endosomal membranes in the mast cells (By similarity).	
28	P0DQZ5	Mastoparan-VT3	60	The synthetic peptide shows antimicrobial activities against Gram-negative bacteria (but not against all strains tested), Gram-positive bacteria (all strains tested) and the fungi C.albicans and C.parapsilosis.Exhibits moderate hemolytic activity (25% at 100 μg/mL) against washed human erythrocytes.	*Vespa tropica* (Greater banded hornet) (Sphex tropica)
29	P0DQZ6	Mastoparan-VT4	60	The synthetic peptide shows antimicrobial activities against Gram-negative bacteria (but not against all strains tested), Gram-positive bacteria (not all strains tested) and the fungi C.albicans and C.parapsilosis. Exhibits little hemolytic activity against washed human erythrocytes.	*Vespa tropica* (Greater banded hornet) (Sphex tropica)
30	P0DQZ7	Mastoparan-VT5	60	The synthetic peptide shows weak antimicrobial activities against a few Gram-positive bacteria (only 2 on the 11 strains tested) and the fungus C.albicans. Does not show activity against all the Gram-negative bacteria tested. Exhibits little hemolytic activity against washed human erythrocytes.	*Vespa tropica* (Greater banded hornet) (Sphex tropica)
31	P0DQZ8	Mastoparan-VT6	60	The synthetic peptide shows antimicrobial activities against Gram-negative bacteria (but not against all strains tested), Gram-positive bacteria (all strains tested) and the fungi C.albicans and C.parapsilosis. Exhibits little hemolytic activity against washed human erythrocytes.	*Vespa tropica* (Greater banded hornet) (Sphex tropica)

32	P0DQZ9	Mastoparan-VT7	58	The synthetic peptide shows antimicrobial activities against Gram-negative bacteria (but not against all strains tested), Gram-positive bacteria (all strains tested) and the fungi C.albicans (but not C.parapsilosis). Exhibits little hemolytic activity against washed human erythrocytes.	*Vespa tropica* (Greater banded hornet) (Sphex tropica)
33	P0DRA2	Mastoparan-VB2 (MP-VB2)	60	Antimicrobial peptide. Shows activity against both Gram-positive and -negative bacteria, as well against fungi. Also promotes moderate mast cell degranulation. Does not show hemolytic activity on rabbit and human erythrocytes (By similarity).<br>Its mast cell degranulation activity may be related to the activation of G-protein coupled receptors in mast cells as well as interaction with other proteins located in cell endosomal membranes in the mast cells (By similarity).	*Vespa bicolor* (Black shield wasp)
34	P17238	Mastoparan (Histamine-releasing peptide I) (HR-1) (HR-I) (HR1)	60	Mast cell degranulating peptide. Its mast cell degranulation activity may be related to the activation of G-protein coupled receptors in mast cells as well as interaction with other proteins located in cell endosomal membranes in the mast cells (By similarity).<br>Has a membranolytic activity on human glioblastoma multiforme cells (brain tumor cells) that leads to cell necrosis.	*Vespa orientalis* (Oriental hornet)
35	P68408	Melittin (MEL) (MLT)	70	Main toxin of bee venom with strong hemolytic activity and antimicrobial activity. It has enhancing effects on bee venom phospholipase A2 activity. This amphipathic toxin binds to negatively charged membrane surface and forms pore by inserting into lipid bilayers inducing the leakage of ions and molecules and the enhancement of permeability that ultimately leads to cell lysis. It acts as a voltage-gated pore with higher selectivity for anions over	*Vespa magnifica* (Hornet)

				cations. The ion conductance has been shown to be voltage-dependent. Self-association of melittin in membranes is promoted by high ionic strength, but not by the presence of negatively charged lipids. In vivo, intradermal injection into healthy human volunteers produce sharp pain sensation and an inflammatory response. It produces pain by activating primary nociceptor cells directly and indirectly due to its ability to activate plasma membrane phospholipase A2 and its pore-forming activity.	
36	P68409	Melittin (MEL) (MLT)	70	Main toxin of bee venom with strong hemolytic activity and antimicrobial activity. It has enhancing effects on bee venom phospholipase A2 activity. This amphipathic toxin binds to negatively charged membrane surface and forms pore by inserting into lipid bilayers inducing the leakage of ions and molecules and the enhancement of permeability that ultimately leads to cell lysis. It acts as a voltage-gated pore with higher selectivity for anions over cations. The ion conductance has been shown to be voltage-dependent. Self-association of melittin in membranes is promoted by high ionic strength, but not by the presence of negatively charged lipids. In vivo, intradermal injection into healthy human volunteers produce sharp pain sensation and an inflammatory response. It produces pain by activating primary nociceptor cells directly and indirectly due to its ability to activate plasma membrane phospholipase A2 and its pore-forming activity.	*Vespa velutina nigrithorax* (Hornet)
37	P0DRA7	Mastoparan MP	14	Antimicrobial peptide. Has activity against both Gram-positive and -negative bacteria (B.subtilis (MIC=9 uM),	*Mischocyttarus phthisicus* (Paper wasp*) (Vespa phthisica)*

				E.coli (MIC=65 uM)). Shows mast cell degranulation activity (EC50=15-26 uM). Has low hemolytic activity (IC50=100 uM).<br>Its mast cell degranulation activity may be related to the activation of G-protein coupled receptors in mast cells as well as interaction with other proteins located in cell endosomal membranes in the mast cells (By similarity).	
38	S4S2C7	Mastoparan-AF (MP-AF)	62	Antimicrobial and mast cell degranulating peptide.<br>Has broad spectrum antibacterial activity against both Gram-positive and Gram-negative bacteria (S.aureus MIC=16-32 µg/ml, S.xylosus MIC=1.5 µg/ml, S.alactolyticus MIC=8 µg/ml, C.koseri MIC=4 µg/mL, E.coli MIC=4-32 µg/ml, K.pneumoniae MIC=32 µg/ml, P.aerugiosa MIC=96 µg/mL, S.choleraesuis MIC=16 µg/mL, S.typhimurium MIC=32 µg/mL, V.parahamelytics MIC=16 µg/mL).<br>Is also active on multi-antibiotic resistant hemolytic E.coli O157:H7.<br>Acts by affecting membrane permeability.<br>On E.coli O157:H7, acts through multiple membrane disruption patterns, including large perforations (full opening) at apical ends (hollow tubes), vesicle budding, forming dents, and membrane corrugation and invagination leading to irregular pits or pores.<br>Exerts 40% lower membrane permeabilization activities on E.coli O157:H7 than on the non-pathogen E.coli BL21.<br>Shows little hemolytic activities on sheep, chicken and human erythrocytes, but with a higher activity on chicken erythrocytes.	*Vespa affinis* (Lesser banded hornet)

				Its mast cell degranulation activity may be related to the activation of G-protein coupled receptors in mast cells as well as interaction with other proteins located in cell endosomal membranes in the mast cells (By similarity).	
39	S4S3F6	Mastoparan-V (MP-V)	60	Antimicrobial and mast cell degranulating peptide. Has broad spectrum antibacterial activity against both Gram-positive and Gram-negative bacteria (S.aureus MIC=32-64 µg/mL, S.xylosus MIC=3 µg/mL, S.alactolyticus MIC=16 µg/mL, C.koseri MIC=4 µg/mL, E.coli MIC=8 µg/mL, K.pneumoniae MIC=64 µg/mL, P.aerugiosa MIC=256 µg/mL, S.choleraesuis MIC=32 µg/mL, S.typhimurium MIC=32 µg/mL, V.parahamelytics MIC=32 µg/mL). Affects membrane permeability of E.coli. Shows hemolytic activities on sheep, chicken and human erythrocytes.<br>Its mast cell degranulation activity may be related to the activation of G-protein coupled receptors in mast cells as well as interaction with other proteins located in cell endosomal membranes in the mast cells (By similarity).	*Vespa velutina flavitarsus* (Asian hornet)
40	S4S3G3	Mastoparan-D (MP-D)	60		*Vespa ducalis* (Black-tailed hornet)
41	Q7M3V3	Orientotoxin-2 (EC 3.1.1.4) (Orientotoxin II) (Phospholipase A2)	139		*Vespa orientalis* (Oriental hornet)
42	Q7M3V4	Orientotoxin-1 (EC 3.1.1.5) (Lysophospholipase) (Orientotoxin I)	152		*Vespa orientalis* (Oriental hornet)
43	P0DMB5	Phospholipase A1 2 (PLA1) (EC 3.1.1.32) (Allergen Ves a 1.02) (Vespapase) (allergen Vesp a 1)	334		*Vespa affinis* (Lesser banded hornet)

44	P0DMB6	Phospholipase A1 verutoxin-1 (PLA1) (VT-1) (EC 3.1.1.32) (EC 3.1.1.5)	23		*Vespa velutina* (Asian yellow-legged hornet)
45	P0DMB7	Phospholipase A1 verutoxin-2a (PLA1) (VT-2a) (EC 3.1.1.32) (EC 3.1.1.5)	25		*Vespa velutina* (Asian yellow-legged hornet)
46	P0DMB8	Phospholipase A1 verutoxin-2b (PLA1) (VT-2b) (EC 3.1.1.32) (EC 3.1.1.5)	25		*Vespa velutina* (Asian yellow-legged hornet)
47	A0A0M3KKW3	Phospholipase A1 (vPLA1) (EC 3.1.1.32)	300		*Vespa basalis* (Hornet)
48	P0DPT0	Phospholipase A1 VesT1.02 (EC 3.1.1.32)	301		*Vespa tropica* (Greater banded hornet) (Sphex tropica)
49	P0DMB4	Phospholipase A1 1 (PLA1) (EC 3.1.1.32) (Allergen Ves a 1.01) (Vespapase) (allergen Vesp a 1)	334		*Vespa affinis* (Lesser banded hornet)
50	P0CH47	Probable phospholipase A1 magnifin (PLA1) (EC 3.1.1.32)	337	Catalyzes the hydrolysis of phosphatidylcholine with phospholipase A1 activity (By similarity); May act as an allergen and induce hemolytic activity (By similarity); In vivo, induces dose-dependent platelet aggregation (nanomolar concentration) and induces thrombosis	*Vespa magnifica* (Hornet)
51	P0CH87	Phospholipase A1 (PLA1) (EC 3.1.1.32) (allergen Vesp c 1)	301		*Vespa crabro* (European hornet)
52	Q6Q249	Phospholipase A1 4 (PLA1 4) (EC 3.1.1.32) (allergen Pol d 1)	316		*Polistes dominula* (European paper wasp) *(Vespa dominula)*
53	Q6Q250	Phospholipase A1 3 (PLA1 3) (EC 3.1.1.32) (allergen Pol d 1)	316		*Polistes dominula* (European paper wasp) *(Vespa dominula)*
54	Q6Q251	Phospholipase A1 2 (PLA1 2) (EC 3.1.1.32) (allergen Pol d 1)	316		*Polistes dominula* (European paper wasp) *(Vespa dominula)*

55	Q6Q252	Phospholipase A1 1 (PLA1 1) (EC 3.1.1.32) (allergen Pol d 1)	337		*Polistes dominula* (European paper wasp) *(Vespa dominula)*
56	C0HLL3	Phospholipase A1 (PLA1) (EC 3.1.1.32) (Vesp v 1)	304		*Vespa velutina* (Asian yellow-legged hornet)
57	D1MEJ7	Putative insulin/insulin-like peptide binding protein	293		*Eumenes pomiformis* (Potter wasp) *(Vespa pomiformis)*
58	D1MEJ9	Putative metallopeptidase	125		*Eumenes pomiformis* (Potter wasp) *(Vespa pomiformis)*
59	D1MEK0	Putative metallopeptidase	102		*Eumenes pomiformis* (Potter wasp) *(Vespa pomiformis)*
60	D1MEK1	Putative metallopeptidase	129		*Eumenes pomiformis* (Potter wasp) *(Vespa pomiformis)*
61	D1MEK2	Putative tyrosine 3/tryptophan 5-monooxygenase	141		*Eumenes pomiformis* (Potter wasp) *(Vespa pomiformis)*
62	Q7YWA7	Secapin	77	Nontoxic peptide.	*Vespa magnifica* (Hornet)
63	Q7YWA8	Secapin	77		*Vespa velutina nigrithorax* (Hornet)
64	D1MEI7	Venom peptide 2a (EpVP2a) (VP2a) (Eumenine mastoparan VP2a)	63		*Eumenes pomiformis* (Potter wasp) *(Vespa pomiformis)*
65	A0SPI1	Vespid chemotactic peptide 5h (VCP 5h)	65	Shows antimicrobial activity against the Gram-negative bacteria E.coli ATCC 25922 (MIC=30 µg/mL), the Gram-positive bacteria S.aureus ATCC 2592 (MIC=5 µg/mL) and the fungus C.albicans ATCC 2002 (MIC=25 µg/mL) (PubMed:17573088); Acts as a mast cell degranulating peptide (By similarity); Its mast cell degranulation activity may be related to the activation of G-protein coupled receptors in mast cells as well as interaction with other proteins located in cell endosomal membranes in the	*Vespa magnifica* (Hornet)

				mast cells (By similarity); Induces the chemotaxis of neutrophils (By similarity).	
66	P0DRA0	Vespid chemotactic peptide VT1 (VCP-VT1) [Cleaved into: Vespid chemotactic peptide VT2 (VCP-VT2)]	65	Vespid chemotactic peptide VT1<br>Antimicrobial peptide with activities against Gram-negative bacteria, Gram-positive bacteria and the fungi C.albicans and C.parapsilosis; Exhibits little hemolytic activity against washed human erythrocytes; Acts as a mast cell degranulating peptide (By similarity); Its mast cell degranulation activity may be related to the activation of G-protein coupled receptors in mast cells as well as interaction with other proteins located in cell endosomal membranes in the mast cells (By similarity); Induces the chemotaxis of neutrophils (By similarity).<br>Vespid chemotactic peptide VT2<br>Antimicrobial peptide with activities against Gram-negative bacteria, Gram-positive bacteria and the fungi C.albicans and C.parapsilosis; Exhibits little hemolytic activity against washed human erythrocytes; Acts as a mast cell degranulating peptide (By similarity); Induces the chemotaxis of neutrophils (By similarity).	*Vespa tropica* (Greater banded hornet) (Sphex tropica)
67	P0DRA3	VESP-VB1	65		*Vespa bicolor* (Black shield wasp)
68	D1MEI8	Venom peptide 2b (EpVP2b) (VP2b) (Eumenine mastoparan VP2b)	63		*Eumenes pomiformis* (Potter wasp) *(Vespa pomiformis)*
69	D1MEJ3	Venom peptide 6 (EpVP6) (VP6) (Protonectin VP6)	62		*Eumenes pomiformis* (Potter wasp) *(Vespa pomiformis)*
70	P0C1M1	Vespid chemotactic peptide 5e (VCP 5e)	13	Mast cell degranulating peptide (By similarity).<br>Shows antimicrobial activity against the Gram-negative bacteria E.coli ATCC 25922 (MIC=30 µg/mL), the Gram-	*Vespa magnifica* (Hornet)

				positive bacteria S.aureus ATCC 2592 (MIC=5 μg/mL) and the fungus C.albicans ATCC 2002 (MIC=25 μg/mL); Has little hemolytic activity; Induces the chemotaxis of neutrophils (By similarity); Its mast cell degranulation activity may be related to the activation of G-protein coupled receptors in mast cells as well as interaction with other proteins located in cell endosomal membranes in the mast cells (By similarity).	
71	P0C1M2	Vespid chemotactic peptide 5f (VCP 5f)	13	Mast cell degranulating peptide (By similarity).<br>Shows antimicrobial activity against the Gram-negative bacteria E.coli ATCC 25922 (MIC=30 μg/mL), the Gram-positive bacteria S.aureus ATCC 2592 (MIC=5 μg/mL) and the fungus C.albicans ATCC 2002 (MIC=25 μg/mL); Has little hemolytic activity; Induces the chemotaxis of neutrophils (By similarity); Its mast cell degranulation activity may be related to the activation of G-protein coupled receptors in mast cells as well as interaction with other proteins located in cell endosomal membranes in the mast cells (By similarity).	*Vespa magnifica* (Hornet)
72	P0C1M3	Vespid chemotactic peptide 5g (VCP 5g)	13	Mast cell degranulating peptide (By similarity).<br>Shows antimicrobial activity against the Gram-negative bacteria E.coli ATCC 25922 (MIC=30 μg/mL), the Gram-positive bacteria S.aureus ATCC 2592 (MIC=5 μg/mL) and the fungus C.albicans ATCC 2002 (MIC=25 μg/mL); Has little hemolytic activity; Induces the chemotaxis of neutrophils (By similarity); Its mast cell degranulation activity may be related to the activation of G-protein coupled receptors in mast cells as well as interaction with other proteins located in cell endosomal membranes in the mast cells (By similarity).	*Vespa magnifica* (Hornet)

73	P0DL40	Vespin	67	Shows contractile activity on isolated ileum smooth muscle.	*Vespa magnifica* (Hornet)
74	P0DMB9	Venom allergen 5 (Antigen 5) (allergen Vesp v 5)	202		*Vespa velutina* (Asian yellow-legged hornet)
75	P0DRA4	VESP-VB2	65		*Vespa bicolor* (Black shield wasp)
76	P0DRB8	Venom dipeptidyl peptidase 4 (EC 3.4.14.5) (Dipeptidylpeptidase IV) (DPPIV) (New Vespv3) (NewVespv3)	776		*Vespa velutina* (Asian yellow-legged hornet)
77	P17231	Vespid chemotactic peptide T (VESCP-T) (Ves-CP-T)	13		*Vespa tropica* (Greater banded hornet) (Sphex tropica)
78	P17232	Vespid chemotactic peptide M (VESCP-M) (Ves-CP-M)	13	Antimicrobial peptide with activities against Gram-negative bacteria, Gram-positive bacteria and the fungi C.albicans and C.parapsilosis (By similarity); Exhibits little hemolytic activity against washed human erythrocytes (By similarity); Mast cell degranulating peptide. Induces the chemotaxis of neutrophils. Its mast cell degranulation activity may be related to the activation of G-protein coupled receptors in mast cells as well as interaction with other proteins located in cell endosomal membranes in the mast cells (By similarity).	*Vespa mandarinia* (Asian giant hornet)
79	P17233	Vespid chemotactic peptide A (VESCP-A)	13		*Vespa analis* (Yellow-vented hornet)
80	P17234	Vespid chemotactic peptide X (VESCP-X) (Ves-CP-X)	13	Antimicrobial peptide against bacteria and fungi (By similarity); Mast cell degranulating peptide. Induces the chemotaxis of neutrophils. Its mast cell degranulation activity may be related to the activation of G-protein coupled receptors in mast cells as well as interaction with	*Vespa xanthoptera* (Japanese yellow hornet) *(Vespa simillima xanthoptera)*

				other proteins located in cell endosomal membranes in the mast cells (By similarity).	
81	P86870	Venom allergen 5 (Antigen 5) (Ag5) (Cysteine-rich venom protein) (CRVP) (allergen Vesp ma 5)	225		*Vespa magnifica* (Hornet)
82	Q7M3T3	Vespakinin-M (Bradykinin-related peptide)	12	Bradykinins are a potent but short-lived agent of arteriolar dilation and increased capillary permeability (By similarity).<br>May target bradykinin receptors (BDKRB). May cause hypotension.	*Vespa mandarinia* (Asian giant hornet)
83	Q7Z269	Venom serine protease (EC 3.4.21.-) (allergen Pol d 4)	277		*Polistes dominula* (European paper wasp) *(Vespa dominula)*
84	D1MEI9	Venom peptide 3 (EpVP3) (VP3)	49		*Eumenes pomiformis* (Potter wasp) *(Vespa pomiformis)*
85	D1MEJ0	Venom peptide 4a (EpVP4a) (VP4a)	52		*Eumenes pomiformis* (Potter wasp) *(Vespa pomiformis)*
86	D1MEJ1	Venom peptide 4b (EpVP4b) (VP4b)	52		*Eumenes pomiformis* (Potter wasp) *(Vespa pomiformis)*
87	D1MEJ2	Venom peptide 5 (EpVP5) (VP5)	56		*Eumenes pomiformis* (Potter wasp) *(Vespa pomiformis)*
88	D1MEJ5	phospholipase A2 (EC 3.1.1.4)	184		*Eumenes pomiformis* (Potter wasp) *(Vespa pomiformis)*
89	P35781	Venom allergen 5.01 (Allergen Vesp c V.01) (Antigen 5-1) (Ag5-1) (Cysteine-rich venom protein) (CRVP) (allergen Vesp c 5.01)	202		*Vespa crabro* (European hornet)

90	P35782	Venom allergen 5.02 (Allergen Vesp c V.02) (Antigen 5-2) (Ag5-2) (Cysteine-rich venom protein) (CRVP) (allergen Vesp c 5.02)	202		*Vespa crabro* (European hornet)
91	P81656	Venom allergen 5 (Antigen 5) (Ag5) (Cysteine-rich venom protein) (CRVP) (allergen Pol d 5)	227		*Polistes dominula* (European paper wasp) *(Vespa dominula)*
92	P81657	Venom allergen 5 (Antigen 5) (Ag5) (Cysteine-rich venom protein) (CRVP) (allergen Vesp m 5)	202		*Vespa mandarinia* (Asian giant hornet)
93	Q7M3T2	Vespakinin-X (Bradykinin-related peptide)	12	Bradykinins are a potent but short-lived agent of arteriolar dilation and increased capillary permeability (By similarity).<br>May target bradykinin receptors (BDKRB). May cause hypotension.	*Vespa xanthoptera* (Japanese yellow hornet) *(Vespa simillima xanthoptera)*
94	Q7M471	Venom protein HR-3	9	Mast cell degranulating peptide.	*Vespa orientalis* (Oriental hornet)
95	D3Y4D7	Vespakinin T	55		*Vespa tropica* (Greater banded hornet) (Sphex tropica)
96	Q0PQX8	Vespakinin-M	55		*Vespa magnifica* (Hornet)
